# Comparative Sensitivity of Canine Swim-Up Sperm Fractions Treated with Antifreeze Protein and Trehalose Following Freeze–Thaw

**DOI:** 10.3390/ani16172664

**Published:** 2026-08-25

**Authors:** Abbas Farshad, Lukas Trzebiatowski, Axel Wehrend

**Affiliations:** Veterinary Clinic for Reproductive Medicine and Neonatology, Justus Liebig-University of Giessen, 35392 Giessen, Germany

**Keywords:** swim up, antifreeze protein, trehalose, sperm cryopreservation

## Abstract

Sperm cryopreservation is widely used in reproductive biotechnology, but the freeze–thaw process often reduces sperm quality by impairing motility, viability, and DNA integrity. This study assessed how antifreeze protein III (AFP III; 1.5 µg/mL) and trehalose (25 mM), alone or combined, influence post-thaw sperm quality in swim-up fractions. After routine semen evaluation, ejaculates were separated into upper, native, and lower fractions and diluted in a Tris–fructose–egg yolk extender containing the additives. Post-thaw analyses covered motility, kinematic parameters, apoptosis, intracellular hydrogen peroxide (H_2_O_2_), mitochondrial activity, lipid peroxidation, viability, and DNA fragmentation. Because all fractions originated from the same ejaculate, differences among native, upper, and lower sperm populations were evaluated using a mixed-effects model with sperm fraction as a fixed effect and dog/ejaculate as a random effect. Native semen showed significant differences among fractions (*p* < 0.05). The upper fraction showed the highest TM and PM, together with clearly superior kinematic performance in VCL, VSL, VAP, and ALH. All these parameters were significantly higher than those measured in the lower fraction (*p* < 0.05). In contrast, the native fraction displayed intermediate values for TM, PM, VCL, VSL, VAP, and ALH, showing a significant difference compared with the upper fraction (*p* < 0.05) but no significant difference relative to the lower fraction (*p* > 0.05). Beat cross frequency (BCF) did not differ among groups (*p* > 0.05). AFP III improved motility and reduced H_2_O_2_-derived ROS (*p* < 0.05), whereas trehalose decreased motility (*p* < 0.05) but increased mitochondrial activity in the lower fraction (*p* < 0.05). After freezing, the upper fraction showed higher lipid peroxidation and DNA fragmentation, while the lower fraction exhibited more apoptosis and reduced viability (*p* < 0.05). Overall, AFP III provided the most consistent protection, and trehalose produced mixed, fraction-dependent effects, confirming that sperm subpopulation characteristics strongly influence freeze–thaw outcomes.

## Simple Summary

Sperm freezing often reduces cell quality, affecting motility, survival, and DNA stability. This study examined how swim-up fractions (upper, native, lower) respond to cryopreservation when treated with AFP III, trehalose, or both. The upper fraction initially showed the strongest motility and kinematic performance, whereas the lower fraction was weakest. After thawing, the upper fraction became more susceptible to lipid peroxidation and DNA fragmentation, while the lower fraction showed higher apoptosis and reduced viability. AFP III consistently improved post-thaw motility and lowered H_2_O_2_-related oxidative stress, whereas trehalose decreased motility but enhanced mitochondrial activity in the lower fraction. Overall, AFP III provided the most reliable protection, and intrinsic differences among sperm subpopulations strongly shaped their freeze–thaw resilience and response to cryoprotective additives.

## 1. Introduction

Sperm cryopreservation is a well-established method for preserving male genetic material for assisted reproductive technologies, including artificial insemination [1]. Despite its widespread use, the freeze–thaw process can substantially impair sperm structure and function, leading to morphological alterations, physiological disruption, and reduced fertilizing capacity [2,3,4,5]. These impairments arise primarily from ice crystal formation, oxidative and mechanical stress, cellular dehydration, and recrystallization, all of which compromise membrane integrity and intracellular organization [6,7,8,9]. To mitigate such cryo-induced damage, cryoprotective agents are routinely incorporated into freezing media, where they help reduce low-temperature-associated cellular and molecular injury [10,11]. To mitigate such cryo-induced damage, cryoprotective agents are routinely incorporated into freezing media, where they help reduce low-temperature-associated cellular and molecular injury [3,4,5,6,7,8,9,10,11,12]. Complementary to chemical protection, sperm selection techniques such as swim-up are widely applied prior to freezing to enhance sample quality and improve post-thaw performance. Through active migration into an upper medium layer, swim-up enriches motile and morphologically superior spermatozoa, yielding fractions with higher motility, improved kinematic profiles, and reduced oxidative stress compared with unselected populations. This method is extensively used in both human and animal reproduction and consistently improves sperm quality, fertilization potential, and freeze–thaw resilience [12,13,14,15,16].

Antifreeze proteins (AFPs) have emerged as promising additives to improve cryopreservation outcomes, with studies reporting enhanced post-thaw sperm quality [3,4,17,18,19]. These ice-binding proteins are found across diverse organisms, including bacteria, fungi, crustaceans, insects, microalgae, and fish, and are classified into types I–III based on structure [20,21,22]. AFPs act by inhibiting ice nucleation and recrystallization, stabilizing membranes, reducing leakage, and inducing thermal hysteresis, thereby helping maintain osmotic balance during freezing [20,23]. Their effects are concentration-dependent, as high levels may induce cytotoxicity, whereas lower concentrations improve sperm quality and cryosurvival [3,4,24,25,26,27]. Among AFP types, AFP III has shown the most consistent benefits across species, highlighting its potential for cryopreservation applications [16,17,18,19].

On the other hand, the protective effect of semen extenders during cryopreservation is enhanced by non-permeable disaccharides, which consistently improve post-thaw sperm survival across species [9,28,29,30]. These sugars create an osmotic gradient that promotes controlled dehydration, reduces intracellular water content, and limits ice crystal formation during freezing [9,12,29,30,31]. They also stabilize cell volume, thereby decreasing membrane fragility and cryo-induced structural damage [9,29,30,32,33]. Among them, trehalose shows the strongest and most consistent cryoprotective effects in goat, ram, buffalo, bull, and rabbit sperm [29,30,34,35,36,37,38]. Structurally, it is a non-reducing disaccharide of two D-glucose units linked by an α,1-1 bond, acting as both an osmolyte and energy source during freezing [29,39]. Its ability to generate a hypertonic environment before freezing promotes controlled dehydration and prevents intracellular ice formation and associated mechanical damage [9,30,40]. Trehalose also associates with membrane phospholipids during freeze–thaw, promoting lipid bilayer reorganization, enhancing membrane fluidity, and lowering the lipid phase transition temperature under dehydration [9,30]. This promotes formation of a stable glass-like matrix that preserves membrane structure at low hydration levels. Experimental membrane models further show that trehalose inhibits lipid mixing and membrane fusion, highlighting its role in maintaining structural stability during water loss [9,30,40]. Functionally, trehalose improves post-thaw sperm quality across species, enhancing motility and survival in goats [9], improving motility and membrane integrity in rams [34,41], and reducing chromatin damage while increasing post-thaw motility in porcine sperm [42].

Although swim-up selection, AFP III, and trehalose have each been studied in the context of sperm cryopreservation, current research does not clarify how these cryoprotective strategies perform within physiologically distinct sperm subpopulations. Most studies evaluate whole ejaculates, overlooking the substantial heterogeneity in motility, oxidative status, membrane stability, and mitochondrial function. This gap limits understanding of whether cryoprotectants act uniformly across spermatozoa or whether their efficacy depends on intrinsic sperm quality. Furthermore, no existing work has systematically compared AFP III and trehalose, alone or in combination, within clearly defined high- and low-quality sperm fractions obtained by swim-up, despite evidence that these additives target different mechanisms of cryodamage. AFP III primarily inhibits ice nucleation and recrystallization, while trehalose acts as a non-permeable disaccharide that promotes controlled dehydration, stabilizes membrane phospholipids, and reduces intracellular ice formation. The absence of integrated studies leads to ambiguous research questions and limits the ability to optimize cryoprotective strategies for specific sperm populations. Therefore, the aim of this study was to characterize high- and low-quality canine sperm subpopulations obtained by swim-up and to evaluate the cryoprotective effects of AFP III (1.5 µg/mL), selected from an effective range of 1.0–2.0 µg/mL [3,4], applied alone or in combination with trehalose. Post-thaw assessments included motility and kinematic parameters, lipid peroxidation, mitochondrial activity, hydrogen peroxide production, viability, apoptosis, necrosis, and DNA integrity, enabling a comprehensive evaluation of cryostability in both sperm fractions.

## 2. Material and Methods

### 2.1. Ethical Approval

Ethical approval for the use of semen samples was granted by the local ethics authority through the Animal Welfare Office of Justus Liebig University Giessen (approval no. kTV 11–2018; approved on 1 October 2024).

### 2.2. Semen Handling and Cryopreservation Methodology

This study was carried out at the Veterinary Clinic for Reproductive Medicine and Neonatology, Justus-Liebig University Giessen, Germany, to assess the breeding potential of fertile male dogs through comprehensive semen evaluation. Eleven ejaculates were collected from eleven clinically healthy dogs of various breeds (Golden Retrievers n = 3, Bernese Mountain Dog, Boxers n = 2, Rhodesian Ridgeback, Collies n = 2, German Shepherd, Great Dane), with a mean age of 5 ± 1.5 years. Semen was obtained via manual penile stimulation, yielding three ejaculate fractions; only the second fraction, characterized by its high sperm concentration, was used for all subsequent analyses. Samples were transported immediately to the laboratory and maintained at 37 °C during initial handling. Each ejaculate was evaluated individually, and only samples exhibiting ≥70% progressive motility and ≥70% morphologically normal spermatozoa were included. Prior to cryopreservation, swim-up separation was performed, and both the upper motile-enriched and the lower residual swim-up fractions were collected separately and processed independently.

**Figure 1:**
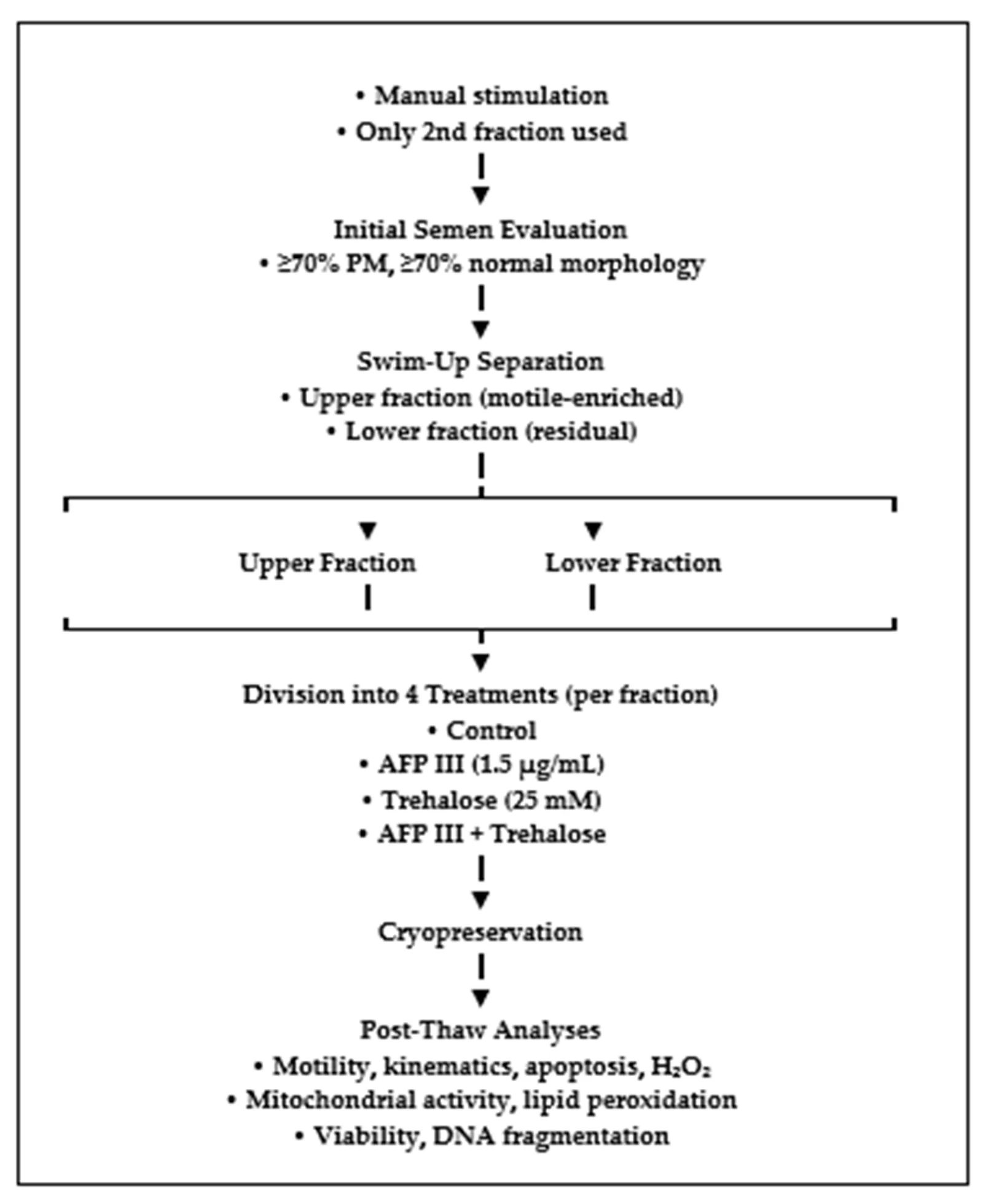
Overview of the Ejaculate Processing, Swim-Up Fractionation, Treatment Allocation, and Cryopreservation Workflow.

Eligible ejaculates were assigned to four treatment groups. A Tris-based extender was prepared using 3.786 g Tris, 2.172 g citric acid, and 1 g fructose dissolved in 100 mL double-distilled water, supplemented with 5% glycerol and 5% egg yolk, yielding a final sperm concentration of ~50 × 10^6^ sperm/mL [3,4,5]. The control group received only the basic extender. Experimental groups were supplemented with antifreeze protein III (AFP III; 1.5 µg/mL; CSB-EP360877FGV, Hoelzel Biotech, Cologne, Germany), trehalose (25 mM; T9449, Sigma-Aldrich, Darmstadt, Germany), or a combination of both. Each treatment was replicated eleven times. After dilution, semen was loaded into 0.25 mL straws, sealed, and equilibrated at 4 °C for 2 h. Freezing was initiated by exposing straws to nitrogen vapor 4–5 cm above liquid nitrogen for 10 min, followed by full immersion for long-term storage (4 weeks). For analysis, straws were thawed individually at 37 °C for 60 s (Figure 1).

### 2.3. Swim-Up Sperm Preparation

Swim-up fractions were generated using a modified density-based migration technique. A 15 mL Falcon tube was prepared with 1 mL of solid polymer in the conical tip to create a flat sedimentation surface. Human Tubal Fluid (HTF) with 0.5% BSA served as the medium. Ejaculates were diluted 1:2 in HTF and centrifuged at 400× *g* for 10 min, allowing sperm and debris to sediment. After removing the supernatant, 1 mL residual fluid remained. Tubes were incubated at 36 °C for 30 min, enabling progressively motile sperm to migrate upward while immotile sperm remained at the base. The upper 0.5 mL motile-enriched fraction was collected gently. Sperm concentration and motility in native ejaculates and swim-up fractions were assessed using a Makler chamber and CASA (Hamilton-Thorne, Beverly, MA, USA) [43].

### 2.4. Sperm Motility Assessment

Motility parameters were quantified using AndroVision™ (Minitüb GmbH & Co, Tiefenbach, Germany) following canine-specific manufacturer guidelines. Five microliters of semen were placed in a pre-warmed 20 µm chamber slide and covered with a coverslip. Video acquisition occurred at 25 fps, and ≥10 microscopic fields were analyzed to ensure ≥200 sperm per sample. The CASA system generated the following motility and kinematic parameters: total motility (TM, %), progressive motility (PM, %), average path velocity (VAP, μm/s), straight-line velocity (VSL, μm/s), curvilinear velocity (VCL, μm/s), straightness (STR = VSL/VAP, µm/s), linearity (LIN = VSL/VCL, µm/s), amplitude of lateral head displacement (ALH, μm), beat cross frequency (BCF, Hz), and wobble (WOB = VAP/VCL, µm/s).

### 2.5. Assessment of Apoptotic Parameters

Apoptotic status was quantified by flow cytometry using Annexin V/PI staining to detect phosphatidylserine externalization. Spermatozoa washed twice in cold BioLegend staining buffer (Cat. No. 420201, San Diego, CA, USA) and resuspended in Annexin V-binding buffer (Cat. No. 422201, San Diego, CA, USA) at 1 × 10^6^ cells/mL. A 100 µL aliquot was incubated with 5 µL FITC-Annexin V (Cat. No. 640905, San Diego, CA, USA) for 15 min at 20–22 °C in the dark, followed by 5 µL propidium iodide (PI; Cat. No. 421301, San Diego, CA, USA) and an additional 15 min incubation. Prior to acquisition, samples were diluted with 400 µL binding buffers, and a minimum of 10,000 events per sample were recorded. Annexin V−/PI+ spermatozoa were detected but not analyzed as a separate category; consistent with previous studies [3,4,5], they were included within the late apoptotic/necrotic population (Annexin V+/PI+).

Flow cytometry was performed on a CytoFLEX system (Beckman Coulter, Krefeld, Germany) operated at low flow rate. Daily calibration with fluorescent beads ensured instrument stability. Compensation was applied using unstained, FITC-only, and PI-only controls. FITC was excited at 488 nm and detected with a 525/40 nm bandpass filter; PI was excited at 561 nm and detected with a 610/20 nm filter. Data was acquired and analyzed in CytExpert 2.7. A structured gating strategy was used: FSC/SSC (Forward/Side Scatter) parameters isolated singlet sperm and excluded debris and aggregates. Fluorescence profiles then distinguished three populations: viable sperm (Annexin V−/PI−), early apoptotic sperm (Annexin V+/PI−), and late apoptotic or necrotic sperm (Annexin V+/PI+) [3,4,5].

### 2.6. Hydrogen Peroxide and Mitochondrial Activity Assessment

Hydrogen peroxide (H_2_O_2_) levels were assessed using the fluorescent probe 2′,7′-dichlorofluorescein diacetate (DCFH-DA; CAS No.: 4091-99-0, Merck KGaA, Darmstadt, Germany). Semen samples were first diluted in phosphate-buffered saline (PBS; P4474 Merck KGaA, Darmstadt, Germany) to a final concentration of 5 × 10^6^ sperm/mL. Thereafter, 25 μL of DCFH-DA was added to 1 mL of the sperm suspension, followed by incubation at 20–22 °C for 40 min. After incubation, samples were centrifuged to remove the supernatant, and the resulting pellet was resuspended in PBS at the same concentration for subsequent analysis. The DCFH-DA stock solution was 10 mM, yielding a final working concentration of 250 µM in the assay [3,4,5].

Mitochondrial membrane potential (MMP) was evaluated using Rhodamine 123 (R123; Cat. No. 83702, Sigma-Aldrich, St. Louis, MO, USA) in combination with propidium iodide (PI; 0.01 mg/mL, Cat. No. 537060, Sigma-Aldrich) as previously described [44]. For MMP analysis, 300 μL of semen was incubated with 10 μL of R123 (0.01 mg/mL) for 20 min at 20–22 °C in the dark. Following incubation, samples were centrifuged at 500× *g* for 3 min and resuspended in 500 μL of Tris buffer. Subsequently, 10 μL of PI (1 μg/mL) was added immediately prior to flow cytometric analysis. Spermatozoa exhibiting R123-positive and PI-negative staining were considered to possess active mitochondria.

### 2.7. Lipid Peroxidation and DNA-Fragmentation Assay

Lipid peroxidation was quantified by measuring malondialdehyde (MDA) levels using the thiobarbituric acid (TBA, CAS-No.: 504-17-6, Merck, Darmstadt, Germany) reaction method [45]. One milliliter of semen was mixed with 2 mL of a trichloroacetic acid (TCA, CAS-No.: 76-03-9, Merck, Darmstadt, Germany) reagent containing 0.375% (*w*/*v*) TBA, 15% (*w*/*v*) TCA, and 0.25 N hydrochloric acid (HCl, CAS-No.: 7647-01-0, Merck, Darmstadt, Germany). The mixture was heated in a water bath at 100 °C for 15 min to allow chromogen formation, then cooled to room temperature. After centrifugation at 1200× *g* for 15 min, the absorbance of the supernatant was measured at 535 nm using a Hitachi U-2001 spectrophotometer (Hitachi, Tokyo, Japan). MDA concentrations were calculated using the specific absorption coefficient (1.56 × 10^5^ L/mol/cm) and expressed as nmol/mL. Before analysis, upper- and lower-layer sperm fractions were adjusted to identical sperm concentrations to ensure that MDA values reflected lipid peroxidation per unit volume rather than differences in cell density or residual extracellular components.

Sperm DNA fragmentation was evaluated using the APO-DIRECT™ TUNEL assay kit (BD Biosciences, Cat. No. 556381, San Jose, CA, USA) following the manufacturer’s protocol with minor adaptations. Frozen–thawed sperm samples were washed in PBS (pH 7.4) and centrifuged at 300× *g* for 5 min to remove debris. Cells were then fixed by gradual addition of 70% ethanol under vortexing and stored at −20 °C for at least 24 h. After fixation, samples were rehydrated with two PBS washes. For labeling, 1 × 10^6^ sperm cells were incubated with a TUNEL reaction mixture containing 45 μL FITC-dUTP labeling solution and 5 μL TdT enzyme for 60 min at 37 °C in the dark. Samples were subsequently washed twice with PBS. For counterstaining, propidium iodide (PI, 0.5 mg/mL) and RNase A (0.1 mg/mL) were added, followed by incubation for 30 min at room temperature in the dark. Flow cytometric analysis was performed on a FACSCalibur™ system (BD Biosciences) using a 488 nm excitation laser, acquiring ≥10,000 events per sample. FITC fluorescence was detected in the FL1 channel (525 ± 15 nm), and PI in FL2 (575 ± 13 nm). Appropriate controls included a negative control (without TdT enzyme) and a positive control (DNase I–treated samples). DNA fragmentation was expressed as DNA Fragmentation Index (DFI), calculated according to the manufacturer’s formula: DFI = (GFTS − GFNC)/GFPC.

### 2.8. Statistical Analysis

Data analysis was performed using mixed-effects modeling in SAS (version 9.1; SAS Institute, Cary, NC, USA) to appropriately account for the non-independence of observations originating from the same ejaculate. Because each ejaculate was split into upper and lower swim-up fractions and each fraction underwent all four cryopreservation treatments, a randomized one-way ANOVA would not adequately represent the experimental structure. Therefore, sperm fraction, treatment, and their interaction were included as fixed effects, while dog/ejaculate was modeled as a random effect to reflect repeated measurements within individuals and to avoid pseudoreplication. When significant fixed effects were detected (*p* < 0.05), Tukey’s HSD test was used for pairwise comparisons. Results are presented as mean ± SD, and different superscript letters indicate statistically significant differences (*p* < 0.05).

## 3. Results

The swim-up procedure produced clear differences in sperm motility and kinematic performance (Table 1). Because native, upper, and lower fractions originate from the same ejaculate, these comparisons were evaluated using a mixed-effects model with sperm fraction as a fixed effect and dog/ejaculate as a random effect. The upper fraction exhibited markedly higher total motility and progressive motility, along with greater velocity-related parameters (VCL, VSL, VAP, ALH) compared with both the native and lower fractions (*p* < 0.05). In contrast, linearity-related parameters (WOB, LIN, STR) were significantly lower in the upper fraction (*p* < 0.05), reflecting its more curved and less constrained movement pattern. No significant differences were detected among fractions for BCF (*p* > 0.05).

Cryopreservation outcomes were influenced by both treatment and sperm fraction (Table 2). Total and progressive motility did not differ between the upper and lower fractions (*p* > 0.05), although AFP III improved motility in the upper fraction, whereas trehalose reduced motility in both fractions.

Among kinematic parameters, the upper fraction showed higher VCL, ALH, and BCF compared with the lower fraction (*p* < 0.05). In contrast, VSL, VAP, WOB, LIN, and STR showed no significant fraction-dependent differences (*p* > 0.05), although trehalose consistently lowered VSL and VAP. Overall, only VCL, ALH, and BCF were significantly affected by sperm fraction (*p* < 0.05), while other kinematic parameters remained unchanged.

Sperm viability and apoptosis-related parameters differed significantly between sperm fractions (Table 3). The upper fraction consistently showed higher viability and lower early and late apoptosis compared with the lower fraction (*p* < 0.05), indicating better membrane stability and reduced apoptotic progression. These differences were observed across all treatments. AFP III produced the lowest apoptosis values within the upper fraction, but the overall pattern remained the same: upper fractions outperformed lower fractions across all endpoints (*p* < 0.05).

Cryopreservation responses differed clearly between the lower- and upper-quality sperm fractions (Table 4). In the lower-quality fraction, all supplemented groups showed reduced H_2_O_2_-positive sperm compared with the control (*p* < 0.05). In contrast, in the upper-quality fraction, only AFP III decreased H_2_O_2_ production (*p* < 0.05), while trehalose and the combined treatment showed no significant effect (*p* > 0.05).

Mitochondrial activity displayed an opposite pattern. In the lower-quality fraction, trehalose and AFP + trehalose significantly increased mitochondrial activity (*p* < 0.001), and AFP III also produced a moderate improvement (*p* < 0.05). However, in the upper-quality fraction, all supplemented groups significantly reduced mitochondrial activity compared with the control (*p* < 0.05).

Overall, AFP III consistently reduced oxidative stress in both fractions, whereas trehalose-based treatments enhanced mitochondrial function only in the lower-quality fraction.

Lipid peroxidation did not differ among treatments within either sperm fraction (*p* > 0.05), although the upper fraction consistently showed higher LPX levels than the lower fraction (*p* < 0.05) (Table 5). DNA integrity followed the same pattern: in the lower fraction, AFP III and AFP + trehalose significantly reduced DNA damage compared with the control (*p* < 0.05), while trehalose produced a moderate but non-significant improvement. In the upper fraction, no treatment-related differences were detected (*p* > 0.05), and DNA fragmentation remained higher overall. Consequently, mean DNA damage was significantly lower in the lower fraction than in the upper fraction (*p* < 0.05), indicating greater genomic stability in lower-quality sperm.

## 4. Discussion

Cryopreservation imposes a complex combination of osmotic, thermal, and oxidative stresses on spermatozoa, disrupting membranes, mitochondria, and chromatin as previously described in humans, livestock, and companion animals [2,3,4,5,9,46,47]. These injuries arise primarily during ice nucleation, crystal growth, and recrystallization, processes known to compromise membrane fluidity, mitochondrial competence, and genomic stability. Although cryoprotectants and antioxidants have been widely applied to counteract these effects [1,3,4,5,9,48], their efficacy varies substantially across species, extenders, and, critically, sperm subpopulations. The present study confirms this variability by demonstrating that sperm quality prior to freezing strongly determines the biological response to cryopreservation and to AFP III and trehalose supplementation. The swim-up procedure produced two distinct sperm populations, consistent with earlier reports describing swim-up as a reliable method for enriching motile, structurally intact spermatozoa [12,13,14,15]. As expected, the upper fraction demonstrated markedly superior functional quality, with higher TM, PM, VCL, VSL, VAP, and ALH. In contrast, the lower fraction exhibited reduced motility and poorer kinematic profiles. These findings reflect the anticipated physiological stratification and identify the upper fraction as the most robust sperm subpopulation. Following cryopreservation, TM and PM values converged between fractions; however, the upper fraction maintained significantly higher VCL, ALH, and BCF, suggesting that its underlying structural and metabolic robustness persists despite an overall decline in motility. Conversely, the lower fraction maintained its compromised status across all endpoints, underscoring its limited cryoresilience. These fraction-dependent differences were reflected in the response to cryoprotective supplementation. AFP III improved post-thaw motility and kinematic parameters exclusively in the upper fraction, producing the highest TM, PM, and BCF values among treatments. This aligns with studies showing that AFPs modulate ice crystal formation and reduce cryoinjury in fish, goats, and dogs [3,4,5,49]. However, AFP III did not enhance motility in the lower fraction, suggesting that its protective action requires intact membranes and functional mitochondria. This interpretation is consistent with the correlation analysis, which showed strong positive associations between motility, velocity, and viability and negative associations between ROS, motility, and mitochondrial function. Similar relationships have been reported in oxidative stress studies across mammalian species [9,44], reinforcing the concept that AFP III can only exert its benefits when baseline cellular integrity exceeds a critical threshold.

Trehalose, despite its established membrane- and mitochondria-stabilizing properties [40,50] and reported benefits in cattle, buffalo, goats, and deer [39,50,51,52], reduced TM and PM in both fractions. This contrasts with most published findings and suggests species- or fraction-specific limitations. In the lower fraction, trehalose significantly increased mitochondrial activity, yet this did not translate into improved motility, indicating dysregulated metabolic activation rather than efficient ATP production. In the upper fraction, all supplemented groups, including trehalose, showed reduced mitochondrial activity, consistent with protective metabolic down-regulation during freezing. These observations align with reports that trehalose’s efficacy depends on extender composition, species-specific membrane architecture, and the physiological status of the sperm [40,53].

Although AFP III and trehalose both modulated oxidative stress, functional improvements did not follow. AFP significantly reduced H_2_O_2_-positive sperm in both fractions, whereas trehalose and AFP + Trehalose produced intermediate reductions. Nevertheless, these changes did not enhance motility or viability, demonstrating that oxidative stress modulation alone cannot restore function when structural damage is advanced. Lipid peroxidation did not differ among treatments but was consistently higher in the upper fraction, suggesting that high-quality sperm may be more vulnerable to freeze–thaw-induced membrane oxidation. Similar patterns have been reported in studies where metabolically active sperm exhibited higher susceptibility to lipid peroxidation [46]. Unexpectedly, genomic stability showed an inverse pattern: DNA fragmentation was significantly lower in the lower fraction across treatments, whereas the upper fraction exhibited consistently higher DNA damage. In the lower fraction, AFP and AFP + Trehalose significantly reduced DNA fragmentation, while trehalose produced intermediate protection. No treatment improved DNA integrity in the upper fraction. These findings contrast with earlier reports of AFP-mediated genomic protection [54] and suggest that high-quality sperm, despite superior motility, may be more susceptible to freeze–thaw-induced genomic instability, possibly due to higher metabolic activity or membrane fluidity. The synergistic reduction in DNA damage observed in the AFP + Trehalose group aligns with studies showing that trehalose enhances the protective effects of other antioxidants [9,54,55], indicating that trehalose may counteract AFP-associated genomic stress.

## 5. Limitations

The interpretation of these findings is influenced by several constraints that shape the scope of the study. To begin with, the relatively small cohort reduces statistical power and may limit the generalizability of the results across the broader canine population. Moreover, breed heterogeneity introduces biological variation in sperm morphology, membrane composition, and cryo-tolerance, which may have contributed to the fraction-dependent differences observed. In addition, the study focuses exclusively on laboratory-based sperm quality parameters and does not incorporate fertility outcomes such as pregnancy rates or litter size, making it difficult to determine how the post-thaw functional values translate into reproductive success. Furthermore, the evaluation was restricted to a single extender system and fixed concentrations of AFP III and trehalose, meaning that alternative formulations or dosages might yield different results. Taken together, these limitations highlight the need for larger, breed-controlled studies that integrate in vivo fertility testing to validate the practical relevance of fraction-specific cryoprotective strategies.

## 6. Conclusions

This study demonstrates that the intrinsic quality of sperm fractions strongly determines their response to cryopreservation and to cryoprotective additives. The upper swim-up fraction exhibited superior motility, kinematic parameters, and viability, along with lower apoptosis before freezing; however, it showed greater susceptibility to lipid peroxidation and DNA fragmentation after thawing. In contrast, the lower fraction displayed reduced functional performance but comparatively lower levels of post-thaw damage. Among the treatments, AFP III (1.5 µg/mL) provided the most consistent protective effect, improving motility and reducing intracellular H_2_O_2_ levels. Trehalose alone exerted mixed, fraction-dependent effects, reducing motility overall while enhancing mitochondrial activity in the lower fraction. These findings further highlight that trehalose does not exert uniform benefits across sperm populations; high-quality sperm may experience osmotic or metabolic stress under trehalose exposure, whereas lower-quality sperm benefit from its membrane-stabilizing properties. Moreover, the pronounced decline in post-thaw motility and velocity observed in both fractions reflects the inherent cryo-sensitivity of canine sperm rather than methodological defects, emphasizing that low CASA-based motility values can still occur even under properly optimized freeze–thaw conditions. In interpreting these outcomes, it is important to recognize that fraction-dependent differences were evaluated within a mixed-effects framework that accounts for repeated measurements within individual dogs, reinforcing that the observed patterns reflect true biological variation rather than statistical artefacts. Overall, these results indicate that both sperm fraction and treatment type significantly influence cryopreservation outcomes, underscoring the need for fraction-specific optimization of freezing protocols in reproductive biotechnology.

## Figures and Tables

**Table 1:** Comparison of motility and kinematic parameters across sperm fractions after the swim-up process of fresh semen.

Parameter	Native	Upper	Lower
TM (%)	86.0 ± 8.7 ^a^	93.7 ± 8.5 ^a^	69.0 ± 22.71 ^b^
PM (%)	72.0 ± 22.0 ^b^	89.0 ± 21.0 ^a^	66.0 ± 20.0 ^b^
VCL (µm/s)	116.7 ± 38.3 ^b^	196.8 ± 71.3 ^a^	97.2 ± 36.0 ^b^
VSL (µm/s)	54.7 ± 22.7 ^ab^	67.7 ± 25.7 ^a^	52.5 ± 22.0 ^b^
VAP (µm/s)	63.8 ± 23.7 ^b^	93.7 ± 30.1 ^a^	61.9 ± 23.4 ^b^
ALH (µm)	0.95 ± 0.32 ^b^	1.72 ± 0.73 ^a^	0.86 ± 0.32 ^b^
BCF (Hz)	12.6 ± 3.0 ^a^	13.6 ± 2.4 ^a^	11.9 ± 3.3 ^a^
WOB (VAP/VCL, µm/s)	0.61 ± 0.10 ^a^	0.48 ± 0.06 ^b^	0.60 ± 0.09 ^a^
LIN (VSL/VCL, µm/s)	0.52 ± 0.10 ^a^	0.35 ± 0.05 ^b^	0.54 ± 0.10 ^a^
STR (VSL/VAP, µm/s)	0.86 ± 0.05 ^a^	0.72 ± 0.06 ^b^	0.86 ± 0.05 ^a^

TM = Total Motility; PM = Progressive Motility; VCL = Curvilinear Velocity; VSL = Straight-Line Velocity; VAP = Average Path Velocity; ALH = Amplitude of Lateral Head Displacement; BCF = Beat Cross Frequency; WOB = Wobble (VAP/VCL); LIN = Linearity (VSL/VCL); STR = Straightness (VSL/VAP). Values are presented as mean ± SD (n = 11). Different superscript letters indicate statistically significant differences between groups (*p* < 0.05). “Upper” refers to sperm collected from the upper layer after the swim-up procedure, whereas “Lower” refers to sperm collected from the lower layer.

**Table 2:** Effects of antifreeze und trehalose on motility kinematic parameters (CASA) of upper and lower sperm fraction after freezing-thawing process.

Parameter	Layer	Control	AFP	Trehalose	AFP + Trehalose	Mean ± SD
TM (%)	Lower	19.4 ± 5.4 ^a^	20.6 ± 7.2 ^a^	15.6 ± 4.0 ^b^	16.3 ± 4.8 ^b^	18.0 ± 2.3 ^A^
TM (%)	Upper	18.3 ± 3.4 ^b^	23.0 ± 9.0 ^a^	13.4 ± 6.0 ^a^	16.7 ± 2.5 ^b^	17.9 ± 3.8 ^A^
PM (%)	Lower	15.3 ± 4.4 ^a^	15.7 ± 5.6 ^a^	11.6 ± 3.1 ^b^	11.9 ± 3.4 ^b^	13.6 ± 1.6 ^A^
PM (%)	Upper	15.4 ± 2.0 ^b^	19.0 ± 8.0 ^a^	11.7 ± 6.1 ^a^	14.1 ± 2.2 ^b^	15.1 ± 2.9 ^A^
VCL (µm/s)	Lower	18.6 ± 7.1 ^a^	17.1 ± 8.4 ^a^	12.3 ± 4.8 ^b^	13.0 ± 5.2 ^b^	15.3 ± 2.9 ^A^
VCL (µm/s)	Upper	16.5 ± 5.9 ^a^	27.3 ± 6.4 ^a^	15.1 ± 6.0 ^a^	13.7 ± 4.4 ^a^	18.2 ± 5.6 ^B^
VSL (µm/s)	Lower	7.3 ± 3.4 ^a^	6.6 ± 3.8 ^a^	3.8 ± 1.6 ^b^	4.0 ± 1.7 ^b^	5.4 ± 1.7 ^A^
VSL (µm/s)	Upper	6.6 ± 2.6 ^a^	10.7 ± 4.2 ^a^	5.6 ± 2.8 ^a^	4.9 ± 2.6 ^a^	6.9 ± 2.0 ^A^
VAP (µm/s)	Lower	9.0 ± 3.7 ^a^	8.4 ± 4.1 ^a^	5.0 ± 1.9 ^b^	5.4 ± 2.0 ^b^	6.9 ± 1.8 ^A^
VAP (µm/s)	Upper	7.9 ± 3.0 ^a^	12.0 ± 4.0 ^a^	7.1 ± 3.0 ^a^	6.1 ± 2.8 ^a^	8.3 ± 2.5 ^A^
ALH (µm)	Lower	0.26 ± 0.07 ^a^	0.24 ± 0.07 ^a^	0.20 ± 0.03 ^b^	0.22 ± 0.05 ^b^	0.23 ± 0.02 ^A^
ALH (µm)	Upper	0.24 ± 0.07 ^a^	0.37 ± 0.03 ^a^	0.24 ± 0.06 ^a^	0.22 ± 0.05 ^a^	0.27 ± 0.05 ^B^
BCF (Hz)	Lower	3.96 ± 2.0 ^a^	3.72 ± 2.1 ^a^	2.63 ± 1.3 ^b^	2.98 ± 1.4 ^b^	3.32 ± 0.6 ^A^
BCF (Hz)	Upper	3.96 ± 1.3 ^a^	6.1 ± 1.9 ^ab^	3.64 ± 1.6 ^b^	2.91 ± 1.4 ^b^	4.15 ± 1.2 ^B^
WOB (µm/s)	Lower	0.47 ± 0.14 ^a^	0.43 ± 0.15 ^a^	0.33 ± 0.12 ^b^	0.36 ± 0.13 ^b^	0.40 ± 0.05 ^A^
WOB (µm/s)	Upper	0.50 ± 0.07 ^a^	0.51 ± 0.03 ^a^	0.44 ± 0.07 ^a^	0.46 ± 0.09 ^a^	0.48 ± 0.03 ^A^
LIN (µm/s)	Lower	0.34 ± 0.13 ^a^	0.32 ± 0.12 ^a^	0.26 ± 0.10 ^b^	0.27 ± 0.11 ^b^	0.30 ± 0.05 ^A^
LIN (µm/s)	Upper	0.42 ± 0.07 ^a^	0.43 ± 0.01 ^a^	0.35 ± 0.07 ^a^	0.36 ± 0.11 ^a^	0.39 ± 0.04 ^A^
STR (µm/s)	Lower	0.80 ± 0.06 ^a^	0.78 ± 0.07 ^a^	0.73 ± 0.06 ^b^	0.74 ± 0.06 ^b^	0.76 ± 0.03 ^A^
STR (µm/s)	Upper	0.83 ± 0.05 ^a^	0.82 ± 0.03 ^a^	0.79 ± 0.06 ^a^	0.79 ± 0.10	0.81 ± 0.02 ^A^

AFP = Antifreeze Protein; TM = Total Motility; PM = Progressive Motility; VCL = Curvilinear Velocity; VSL = Straight-Line Velocity; VAP = Average Path Velocity; ALH = Amplitude of Lateral Head Displacement; BCF = Beat Cross Frequency; WOB = Wobble (VAP/VCL); LIN = Linearity (VSL/VCL); STR = Straightness (VSL/VAP). Different lowercase superscripts within a row indicate significant differences among treatments (*p* < 0.05), whereas uppercase superscripts indicate significant differences between sperm fractions. Values are presented as mean ± SD. The last column shows the overall mean ± SD for each fraction across treatments; different uppercase letters indicate significant differences between fractions (*p* < 0.05; n = 11).

**Table 3:** Impacts of control, AFP, trehalose, and AFP + trehalose treatments on the viability and apoptotic status of upper- and lower-layer spermatozoa following swim-up separation and subsequent freeze–thaw procedures.

Parameter	Treatment	Lower	Upper
Viability (Annexin V^−^/PI^−^)	Control	33.4 ± 14.4 ^b^	34.3 ± 12.7 ^b^
	AFP	34.9 ± 15.3 ^a^	39.0 ± 14.9 ^a^
	Trehalose	30.3 ± 12.5 ^ab^	35.8 ± 16.3 ^ab^
	AFP + Trehalose	32.3 ± 14.1 ^b^	35.4 ± 9.4 ^b^
	Mean ± SD	32.7 ± 2.0 ^B^	36.1 ± 2.1 ^A^
Early apoptosis (Annexin V^+^/PI^−^)	Control	29.5 ± 6.7 ^c^	28.9 ± 10.5 ^c^
	AFP	27.0 ± 9.1 ^a^	23.2 ± 10.5 ^a^
	Trehalose	29.9 ± 8.7 ^b^	23.4 ± 12.4 ^b^
	AFP + Trehalose	28.8 ± 11.2	27.7 ± 9.7 ^b^
	Mean ± SD	28.8 ± 1.1 ^B^	25.8 ± 2.7 ^A^
Late apoptosis (Annexin V^+^/PI^+^)	Control	37.1 ± 13.8 ^c^	36.8 ± 10.9 ^c^
	AFP	38.1 ± 14.7 ^a^	37.8 ± 14.4 ^a^
	Trehalose	39.8 ± 11.4 ^b^	40.8 ± 15.7 ^b^
	AFP + Trehalose	38.9 ± 13.2 ^b^	36.9 ± 10.5 ^b^
	Mean ± SD	38.5 ± 1.1 ^B^	38.1 ± 1.8 ^A^

AFP = Antifreeze Protein; Different lowercase superscript letters within a row indicate statistically significant differences among treatments (*p* < 0.05). Uppercase superscript letters indicate statistically significant differences between sperm layers (lower vs. upper fraction). Values are presented as mean ± SD. The final row for each parameter shows the mean ± SD across all treatments for each fraction, with different uppercase letters ^(A, B)^ indicating significant layer-specific differences. (n = 11).

**Table 4:** Impacts of control, AFP, trehalose, and AFP + trehalose treatments on H_2_O_2_-ROS and mitochondrial activity of upper- and lower-layer spermatozoa following swim-up separation and subsequent freeze–thaw procedures.

Parameter	Treatment	Lower	Upper
H_2_O_2_-ROS (%)	Control	52.5 ± 3.5 ^a^	53.69 ± 18.77 ^a^
	AFP	38.5 ± 6.5 ^b^	33.53 ± 20.09 ^b^
	Trehalose	40.0 ± 10.0 ^b^	42.78 ± 19.33 ^ab^
	AFP + Trehalose	42.5 ± 2.5 ^b^	46.41 ± 20.43 ^ab^
	Mean ± SD	43.4 ± 6.5 ^B^	44.6 ± 8.9 ^A^
Mitochondrial activity (%)	Control	41.5 ± 3.5 ^ab^	49.44 ± 17.54 ^a^
	AFP	54.0 ± 8.5 ^a^	33.96 ± 14.63 ^ab^
	Trehalose	65.0 ± 10.0 ^c^	36.63 ± 15.14 ^b^
	AFP + Trehalose	67.5 ± 7.5 ^c^	32.94 ± 14.62 ^b^
	Mean ± SD	57.0 ± 11.3 ^A^	38.7 ± 7.4 ^B^

AFP = Antifreeze Protein. Values are presented as mean ± SD. Different lowercase superscript letters within a row indicate statistically significant differences among treatments within the same sperm fraction (*p* < 0.05). The final row for each parameter shows the mean ± SD across all treatments for each fraction, with different uppercase letters ^(A, B)^ indicating significant layer-specific differences. (n = 11).

**Table 5:** Impacts of control, AFP, trehalose, and AFP + trehalose treatments on LPX (nmol/mL) and DNA (%) damage of upper- and lower-layer spermatozoa following swim-up separation and subsequent freeze–thaw procedures.

Parameter	Treatment	Lower	Upper
LPX (nmol/mL)	Control	263.45 ± 34.42 ^a^	278.00 ± 41.30 ^a^
	AFP	231.69 ± 56.22 ^a^	256.40 ± 46.80 ^a^
	Trehalose	237.48 ± 35.71 ^a^	263.60 ± 34.80 ^a^
	AFP + Trehalose	243.48 ± 39.47 ^a^	284.60 ± 26.90 ^a^
	Mean ± SD	244.0 ± 13.9 ^B^	270.7 ± 12.0 ^A^
DNA Damage/Fragmentation (%)	Control	31.42 ± 13.32 ^a^	33.10 ± 14.50 ^a^
	AFP	19.43 ± 6.23 ^b^	34.90 ± 13.20 ^a^
	Trehalose	22.45 ± 9.17 ^ab^	30.70 ± 11.20 ^a^
	AFP + Trehalose	17.72 ± 8.54 ^b^	27.90 ± 10.50 ^a^
	Mean ± SD	22.8 ± 6.2 ^B^	31.7 ± 3.1 ^A^

AFP = Antifreeze Protein. Values are presented as mean ± SD. Different lowercase superscript letters within a row indicate statistically significant differences among treatments within the same sperm fraction (*p* < 0.05). Uppercase superscript letters denote significant differences between sperm fractions (lower vs. upper) for the overall mean values. The final row for each parameter shows the mean ± SD across all treatments for each fraction, with different uppercase letters ^(A, B)^ indicating significant layer-specific differences. (n = 11).

## Data Availability

The data generated and analyzed during this study are available from the corresponding author upon reasonable request.
